# Technological Improvement of Brined Black Table Olives Processed Using Two-Phase and Single-Phase Methods Under Slight CO_2_ Pressure and Low Salt Content

**DOI:** 10.3390/foods13233799

**Published:** 2024-11-26

**Authors:** Biagi Angelo Zullo, Gino Ciafardini

**Affiliations:** Department of Agricultural, Environmental and Food Sciences, University of Molise, Via de Sanctis, I-86100 Campobasso, Italy; biagi.zullo@unimol.it

**Keywords:** bacteria, carbon dioxide, fermentation, Leccino, salt, table olives, yeasts

## Abstract

The aim of this work was to study the fermentation of black table olives under slightly pressurized CO_2_ (spCO_2_). The olives were marinated in brine with a low salt content and processed using both the traditional two-phase method and a new single-phase method. SpCO_2_ is a new technical tool, positively tested in previous studies on the production of low-salt table olive, as a third barrier to microbial growth in brine. The tests performed with the cultivar Leccino, using a brine acidified with 0.5% (*w v*^−1^) citric acid and enriched with 0%, 3%, and 6% (*w v*^−1^) NaCl, showed the absence of bacteria and molds in the brine from the first days of incubation. Fermentation was governed by six yeast species, mainly represented by *Candida boidinii* and *Saccharomyces cerevisiae*, with a maximum total number of 6.30 Log CFU mL^−1^ and 4.28 Log CFU mL^−1^ in the brine with 3% and 6% (*w v*^−1^) NaCl, respectively. The best debittering results were obtained when the olives were processed in the presence of spCO_2_ with the single-phase method, using brine with 6% (*w v*^−1^) NaCl, validating the important role played by spCO_2_ in the production of low-salt black table olives.

## 1. Introduction

Table olives are the most important fermented vegetables in the Mediterranean area due to their global economic value. Before consumption, table olives must be processed to reduce their content of oleuropein and its aglycones, which impart a bitter taste to the fruit. Table olives are prepared through three main methods: the Spanish process, in which green olives are treated with lye; the Californian process, which incorporates lye treatment and air oxidation; and the natural-style method, wherein the fruits are brined without undergoing a NaOH treatment and left to ferment until they at least partially lose their bitterness [1,2]. The traditional natural processing of black table olives is carried out in two phases. The first phase includes the fermentation and storage of the fruits for 8–12 months in brine with 8–12% (*w v*^−1^) NaCl in 160–200 L polyvinyl chloride (PVC) barrels or in 20-ton stainless steel silos. The second phase involves the final packaging of the debittered product to be marketed using glass jars or other containers, filled with new brine containing 5–6% (*w v*^−1^) NaCl, acidified with organic acids, and pasteurized [3]. In this study, in addition to the traditional two-phase processing method, we tested for the first time a new method, called the single-phase processing method, in which the fermentation and packaging phases were grouped into a single process. The new processing method allows us to simplify the olive processing and to reduce production costs.

Naturally processed olives are preferred by consumers because they are free from chemical treatments. However, although their nutritional properties are well known, their consumption can generally be limited by their high salt content. Salt consumption is one of the major concerns of the population, and saltiness is one of the attributes included in the sensory evaluation sheet of table olives [4]. According to the World Health Organization (WHO) [5], the recommended daily salt intake should be reduced to <5 g/day of salt for adults, and the European Community reference intake has been set at 2.4 g of NaCl per day [6]. Low salt intake helps reduce blood pressure and the risk of cardiovascular disease [7]. Paltaki et al. [8] highlighted significant interest by consumers in reduced-salt food products, including table olives. The reduction in NaCl content in the brine during olive fermentation and in the final product has attracted the attention of the scientific community in the last decade. Some studies have focused on the use of salt mixtures during the fermentation process and storage of olives in brine [9,10,11]; others have explored the desalination process of the product before packaging [12].

A possible strategy to address the problem could be to improve the current production technology of naturally fermented black table olivesby introducing new obstacles to microbial growth in the brine. In some of our previous studies, we found that slight pressurization with carbon dioxide (spCO_2_) of less than 1 MPa can inhibit or reduce microbial growth in the brine of naturally fermented black table olives with low pH and reduced NaCl concentration [13]. The CO_2_ dissolved in the brine as carbonic acid lowers the pH and simultaneously penetrates the microbial cells, inhibiting their growth [14]. The tests carried out at the pilot plant scale indicated that a spCO_2_ of 1 bar is able to inhibit the growth of bacteria and molds in a brine with reduced salt concentrations [13]. Other research carried out on the fermentation of black table olives with a reduced NaCl content confirmed that the growth of microorganisms in the brine is differently inhibited by spCO_2_ as well as by low pH and salt [15]. On the basis of these first results, it seems that the immobilization in the brine of the CO_2_ released by the fermentation process and the respiration of the fruits can contribute significantly to the improvement of the current production technology of naturally fermented black table olives [16]. However, despite its future prospects, the use of spCO_2_ in the production of table olives is still little studied because it was introduced only recently.

The aim of the present work was to study the natural spCO_2_ fermentation of black table olives, in the presence of low salt content, with the traditional two-phase system and a new single-phase method.

## 2. Materials and Methods

### 2.1. Laboratory Tests on Brine Acidification

Preliminary tests were performed in the laboratory to investigate some technical–scientific aspects related to the acidification of brine with 0.5% (*w v*^−1^) citric acid, reported in the following tests. The tests concerned the influence of the acid pH of brine on the survival of bacteria isolated from the olive carposphere and its interference with some of the current methods of analysis of NaCl in brine.

#### 2.1.1. Survival of Bacteria in Acidified Brine

The test was performed in the laboratory in order to evaluate the survival of total aerobic bacteria (TAB) and lactic acid bacteria (LAB) isolated from the fruits in a brine with low NaCl content (3%, *w v*^−1^) and different pH levels in the absence of spCO_2_. Both types of bacteria came from the same batch of Leccino cultivar olives used in the subsequent tests. Approximately 500 g of olives were placed in a sterile 2000 mL flask, followed by 500 mL of sterile distilled water, and shaken at 200 rpm for approximately 60 min. Finally, 1 mL of sample was serially diluted by a factor of 10 in sterile Ringer’s solution (0.9%, *w v*^−1^ NaCl), and 0.2 mL of each dilution was spread onto specific agar media as described below. LAB grown after 48 h on the de Man, Rogosa, Sharpe (MRS) agar medium, reported below, were verified by phenotypic observation under a light microscope, the catalase test with H_2_O_2_, and Gram staining using the HT90-1-4 Kit (Sigma-Aldrich, St. Louis, MO, USA). Approximately 300 bacterial colonies of TAB and LAB were randomly picked from their respective specific media and transferred separately into two Pyrex tubes containing 9 mL of sterile Ringer’s solution. After vortexing, the O.D._595_ of the two bacterial suspensions was adjusted to 0.940 and used for the inoculation trials of three types of brines with 3% (*w v*^−1^) NaCl without olives. The first brine was acidified with 0.5% (*w v*^−1^) citric acid and showed a pH of 2.32, the second was acidified to a pH of 2.32 using 1 N HCl, and the third was not acidified (pH 7.20). Measures of 9 mL of each brine, sterilized using microfiltration through nitrocellulose filters with a pore size of 0.45 µm (Minisart NML-Sartorius, Göttingen, Germany), were transferred into sterile Pyrex tubes and inoculated separately with 1 mL of the respective bacterial suspension. The test consisted of 3 repetitions. After vortexing, the samples were stored at room temperature for 45 days. The survival of bacteria inoculated in three types of brine was assessed using microbiological analysis of 1 mL of brine samples taken after 1, 2, 7, 15, 30, and 45 days using the selective medium as described below.

#### 2.1.2. NaCl Determination in Acidified Brines

The influence of brine acidification with citric acid on the determination of NaCl concentration was studied in the laboratory using acidified brine samples containing same salt concentrations used in the tests reported below or the same brines neutralized before analysis. Samples of 100 mL of brine with 3% and 6% (*w v*^−1^) NaCl and 0.5% (*w v*^−1^) citric acid were analyzed using titration (Mohr’s method) with 0.1 N AgNO_3_ in neutral solution using K_2_CrO_4_ as an indicator [17]. All brine samples were analyzed directly without any pretreatment or after adjusting the pH to 6.5 using 1 N NaOH solution. The tests were replicated three times.

### 2.2. Processing Tests with the Two- and Single-Phase Method Under spCO_2_

The experiments were conducted under spCO_2_ using the fruits of cv. Leccino (*Olea europea* L.). The olives were harvested at the ripening stage, corresponding to 70% black surface color, from mid-September to mid-October of 2022. After harvesting, the leaves and other inert materials were removed from the olives, and the fruits were washed with tap water, sorted according to size, and finally used in the trials described below.

#### 2.2.1. Traditional Two-Phase Processing Test with spCO_2_ and Different NaCl Contents

In general, the natural processing of black table olives is carried out in two phases. In the first phase, the olives are stored in brine in containers of different sizes, where they undergo the debittering process; in the second phase, they are packaged into smaller containers, covered with new brine, and pasteurized. The traditional two-phase processing method has been technologically improved by introducing spCO_2_ as a new antimicrobial agent. The olives, after being washed with tap water, were placed in 5 L polyethylene terephthalate (PET) demijohn bottles equipped with large self-screw caps. Each demijohn bottle contained 2.6 kg of olives covered with 2.4 L of brine containing 0%, 3%, and 6% (*w v*^−1^) NaCl and acidified with 0.5% (*w v*^−1^) citric acid. The trials were accomplished with three repetitions. All the demijohn bottles were hermetically closed with the screw caps equipped with a 0.5 bar pressure controller device and incubated under spCO_2_ self-produced by the respiratory activity of the fruits and the fermentation by the microbiota. Incubation under spCO_2_ of 0.5 bar occurred for 10 months at 15–17 °C. During the first 5 months of incubation, brine samples were collected at 24 h and 15 days of incubation and every month in an axenic manner after slight loosening of the threatened caps. At six and ten months of incubation, both the brine and olives samples were analyzed. At the end of the incubation period, the second phase of the traditional natural transformation of the black table olives performed under spCO_2_ conditions was applied. After ten months of incubation under spCO_2_ conditions, the Leccino cv. olives were removed from the demijohn bottles and distributed into glass jars. The olives previously marinated in brines with 0%, 3%, and 6% (*w v*^−1^) NaCl and 0.5% (*w v*^−1^) citric acid for 10 months under spCO_2_ condition were carefully washed with water and then divided into glass jars tightly closed with a metal lid with a “click” vacuum indicator device. Each glass jar contained 185 g of olives covered with approximately 130 mL of fresh brine with 6% (*w v*^−1^) NaCl and 0.5% (*w v*^−1^) citric acid. After filling, the glass jars were hermetically sealed, were pasteurized at 85 °C for 20 min, and finally stored in the dark at room temperature. After 2 months of storage, three pasteurized glass jars for each type of treatment were subjected to sensorial analysis, while the rest of the samples collected previously during the first phase of the debittering process were subjected to microbiological, physicochemical, and sensorial analysis.

#### 2.2.2. Single-Phase Processing Trials with spCO_2_ and Different NaCl Contents

The traditional transformation system of naturally fermented black table olives was simplified by merging the olive debittering phase with the packaging phase in a single process using the same containers and the same brine. The laboratory experiments were carried out using the Leccino olives described above. The same batch of fruits used in the previous test was distributed in commercial glass jars with a nominal volume of 314 mL equipped with a metal lid with a “click” vacuum indicator device. Each glass jar contained approximately 190 g of olives covered with 120 mL of brine containing 0%, 3%, and 6% (*w v*^−1^) NaCl and 0.5% (*w v*^−1^) citric acid [3]. For each treatment, 30 tightly closed glass jars were prepared and stored for 10 months at 15–17 °C under the same conditions as the previous test. During incubation, 3 glass jars for each treatment were randomly removed and analyzed at the same times indicated in the previous test. Glass jars opened after analysis were discarded. At the end of the incubation, the jarred olives were pasteurized, stored, and finally analyzed in the same way as the samples from the previous test.

### 2.3. Microbiological Analysis and Yeast Dominance

Microbiological analysis was accomplished using both the brine and the olive flesh in order to quantify the main microbial group (bacteria, yeasts, and molds) involved in the black table olive fermentation. Among the bacteria, the total aerobic bacteria (TAB), total anaerobic bacteria (TANB), lactic acid bacteria (LAB), and enterobacteria were evaluated. The brine was directly analyzed without any pre-treatment, whereas the fruits were treated as follows. The olive samples were first washed separately with sterile distilled water and pitted with a laboratory device. Measures of 2 g of flesh were homogenized for 5 min at 5000 rpm (Turrax model T25; IKA, Milan, Italy) into 25 mL sterile beakers in the presence of 10 mL of sterile Ringer’s solution (0.9% NaCl, *w v*^−1^). Brine and flesh samples were serially diluted by a factor of 10 in sterile Ringer’s solution and spread on specific agar media, as described below. The TAB were enumerated after 24 h of incubation at 30 °C on nutrient agar (CM 0003, Oxoid, Basingstoke, UK) supplemented with 0.05% (*w v*^−1^) cycloheximide (Sigma-Aldrich, St. Louis, MO, USA). The TANB were enumerated on peptone yeast-extract glucose agar (PYGA) medium [18]. The number of bacterial colonies was recorded after 72 h of incubation at 30 °C under anaerobiosis (AnaeroGen and AnaeroJar, Oxoid). The LAB were enumerated using MRS agar medium (Oxoid, Basingstoke, UK) supplemented with 0.02% (*w v*^−1^) cycloheximide (Sigma-Aldrich). The plates were incubated under anaerobiosis at 30 °C for 72 h. Violet Red Bile Glucose (VRBG; Biolife, Milan, Italy) agar was used for enterobacteria, incubated aerobically at 37 °C for 24 h. The number of total yeasts was estimated on malt, yeast, glucose, peptone (MYGP) agar medium [15] supplemented with chloramphenicol (0.1 g L^−1^). The colonies were recorded after incubation at 30 °C for 5 days. The total molds were evaluated after 7 days of incubation at 28 °C on glucose yeast extract agar (GYEA; Oxoid). The microbiological analysis was repeated 3 times for each sample. The results were expressed as Log values of colony-forming units (CFUs) per mL of brine (Log CFU mL^−1^) or per g of flesh (Log CFU g^−1^). The detection limit was established as 10 CFU mL^−1^ or g^−1^. The dominance of yeast species during fermentation was evaluated by transferring approximately 100 randomly selected yeast colonies from each analyzed sample into a series of master cultures. Tests were performed using CHROMagar Candida medium (BBL, cod. 4354093 Heidelberg, Germany) as previously reported [19]. Based on the chromogenic characteristics of the colonies, the presence of pseudohyphae, and the shape and size of the cells, the yeasts were divided into homogenous chromogenic groups. From each homogenous chromogenic group, 5 yeast isolates were randomly selected and subjected to molecular identification at a species level using the procedure previously reported [13].

### 2.4. Physicochemical Parameters

The physicochemical characteristics (pH, titratable acidity, sodium chloride, total polar phenols, CO_2_) of the olive brines and fruits, processed under spCO_2_, were determined as described below. The brine was directly analyzed, whereas the flesh was pre-treated before analysis. The aqueous extract obtained from the fruits according to the previously reported procedure [15] was subjected to chemical analysis for the determination of the NaCl concentration, total polar phenols content, and bitterness index (K_225_). The pH of the brine was evaluated directly on the samples, while that of the olive flesh was evaluated using 2 g of homogenized flesh in 10 mL of distilled water. This parameter was determined via a pH meter using an In Lab Routine Probe (Mettler, Toledo, OH, USA). The titratable acidity of the olive brine was evaluated via titration using the 0.1 N NaOH solution. The equivalence point was detected through potentiometry at pH 8.3. Based on the indications obtained from the preliminary laboratory tests performed on the interference of the brine acidification, the pH of both brine and flesh was corrected to 6.5 with 1 N NaOH solution before the evaluation of the NaCl content. The NaCl content was determined using Mohr’s method: 1 mL of sample was first diluted with 50 mL of distilled water and then titrated with 0.1 N AgNO_3_ using K_2_ CrO_4_ as the indicator [1]. The total polar phenol content of both the brine and olive flesh was evaluated using the Folin–Ciocalteu’s phenol colorimetric method, as described by Ciafardini and Zullo [16]. The K_225_ of the olive flesh was evaluated as described by Ciafardini and Zullo [15]. The phenolic extract obtained from olive flesh using a mixture of methanol and distilled water (80:20, *v v*^−1^) was first diluted with the same extracting mixture and then analyzed with a model 941 Uvikon spectrophotometer at a wavelength of 225 nm (Kontron Instruments, Milan, Italy). K_225_ is expressed as ABS_225_ g^−1^ olive flesh. Each chemical analysis was repeated three times. The density of the brine was evaluated at 20 °C in a 100 mL glass cylinder using a hydrometer equipped with a scale ranging from 1.000 to 1.250 g mL^−1^, with divisions of 0.001 g mL^−1^ (www.paderno.it, accessed on 10 April 2023). The concentration of CO_2_ dissolved in the brine was evaluated on 150 mL samples carefully collected and stored in hermetically sealed bottles, taking care not to leave any headspace between the surface of the liquid and the lid. At the time of analysis, 25 mL of each sample reported above was measured, carefully transferred to a beaker, and titrated using a 0.0454 N solution of anhydrous Na_2_CO_3_, previously dried in an oven at 110 °C. The titration was concluded when the pH of the solution reached the value of 8.3. The remaining part of each brine sample was transferred to a 500 mL plastic vessel where the CO_2_ was released by applying 5 cycles of stirring with a Girmi 400 Watt immersion blender for 1 min, followed by 2 min of rest (https://www.girmi.it, accessed on 10 April 2023). Then, 25 mL of brine was analyzed with the same method as before, and the concentration of free CO_2_ present in the original brine samples was calculated using the difference between the two analyses. The concentration of free CO_2_ was calculated by applying the following formula: CO_2_ (mg L^−1^) = a N 22 1000 V^−1^, where a is the mL of Na_2_CO_3_, N is the normality of the titrant solution, V is the sample volume, and 22 is the equivalent weight of CO_2_ [20].

### 2.5. Packaging and Pasteurization Tests

Packaging concerned only Leccino table olives processed in demijohn bottles according to the traditional two-phase processing method. The olives incubated for ten months in demijohn bottles filled with brine containing 0%, 3%, and 6% (*w v*^−1^) NaCl and acidified with 0.5% (*w v*^−1^) citric acid were removed from their respective containers, washed with potable water, and distributed in commercial glass jars with a nominal volume of 314 mL. Each glass jar contained approximately 190 g olives covered with 120 mL of brine containing 6% (*w v*^−1^) NaCl [3], acidified with 0.5% (*w v*^−1^) citric acid. After filling, the glass jars were hermetically closed with a metal lid with a “click” vacuum indicator device. Ten glass jars were prepared for each treatment. Twenty-four hours after their preparation, the glass jars were analyzed from a microbiological point of view and subjected to pasteurization together with the same number of glass jars from the single-phase processing method mentioned above. Pasteurization was performed at 85 °C for 20 min in accordance with other studies [21,22,23]. During pasteurization in a water bath, the temperature inside the glass jars and the nominal temperature set on the outside of the containers were recorded. After pasteurization, the glass jars were stored at room temperature (15–17 °C) and in the dark for 2 months. At the end of storage, all samples were analyzed as final products from chemical, microbiological, and sensorial perspectives. For each treatment (NaCl concentration) applied to the two-phase and single-phase processing method, three samples were analyzed separately.

### 2.6. Sensory Analysis

Sensory analysis was performed on olives after six and ten months of incubation under spCO_2_ conditions and on olives as the final product. The gustatory attributes related to the odor, saltiness, bitterness, abnormal fermentation, mustiness, and overall quality were evaluated by 10 trained evaluators (five men and five women) with ample experience in the sensory analysis of table olives. The sensory score attributes were as follows: unsatisfactory (1 point), moderate (2 points), good (3 points), and excellent (4 points). All panelists, who were members of the department staff, gave their verbal consent to participate in the test.

### 2.7. Statistical Analysis

All statistical comparisons were first performed using one-way ANOVA omnibus F-Test. When a significant (*p* < 0.05) value of F-statistics was found, differences between means were evaluated using Tukey’s HSD (honestly significant difference) multiple comparison test (*p* < 0.05). Statistical software was used for data processing (Statsoft version 7.0 for Windows, Tulsa, OK, USA).

## 3. Results and Discussion

### 3.1. Tests with Acidified Brine

**Figure 1:**
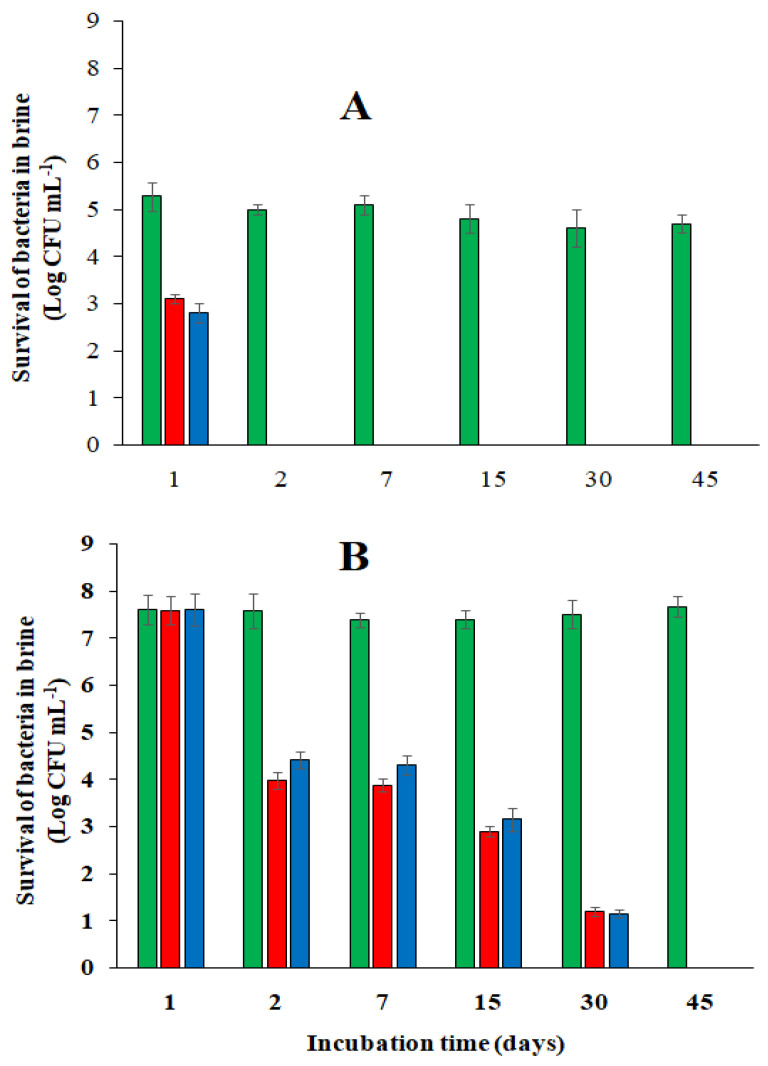
Survival of lactic acid bacteria (**A**) and aerobic bacteria (**B**) in brine without olives, acidified with citric acid (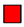), acidified with HCl (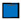), or not acidified (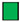).

The traditional processing of black table olives involves several critical issues, including the high salt content, which has the purpose of controlling contaminating microorganisms, especially at the beginning of fermentation, when the pH has not yet reached safety limits. Acidification with citric acid of a brine characterized by a lower salt concentration than is usually used at the beginning of fermentation helps compensate for its lower antimicrobial activity when the values of the self-produced spCO_2_ are still not very consistent. The tests carried out in the laboratory indicated that- in the absence of spCO_2_, acidification of the brine with 0.5% (*w v*^−1^) citric acid inhibits the growth of several groups of bacteria isolated from the olive carposphere. Lactic acid bacteria (LAB) showed greater sensitivity than the others since they survived only 24 h in the acidified brine (Figure 1A). On the contrary, the concentration of total aerobic bacteria (TAB) gradually decreased during the first month of incubation until it reached zero after 45 days of incubation (Figure 1B). A total of 75% of the TAB initially detected in the brine with 3% (*w v*^−1^) NaCl but not acidified with citric acid was able to grow on the VRBG agar medium used to detect the presence of enterobacteria. However, the inhibitory activity does not seem to be strictly related to the type of acid used but to the initial pH of 2.32 produced in the brine after acidification with 0.5% (*w v*^−1^) citric acid or with HCl (Figure 1A,B). The acidification of the brine with citric acid plays a significant role at the beginning of the fermentation of black table olives in the presence of a low salt content because it has a “shock effect” on many microorganisms. From Figure 1, it is possible to note how during a month of incubation the concentration of TAB was reduced by about 85%; however, 48% and 59% of the reduction in the microbial load occurred in the first 48 h of incubation in the brines acidified, respectively, with HCl or citric acid. The evaluation of the NaCl concentration in the brine and in the olive flesh is a significant parameter in the management of the fermentation process of black table olives with low NaCl content. The values of this parameter can be acquired by determining the mineral components by absorption spectrophotometry [12]. However, in practice, analyses performed using potentiometry or titration with AgNO_3_ (Mohr’s method) are very common. A direct analysis of brines acidified with citric acid performed with these latter methods leads to an overestimation of the concentration of NaCl present in the samples. The results of titration tests of two fresh brines prepared with 3% and 6% (*w v*^−1^) NaCl and acidified with 0.5% (*w v*^−1^) citric acid, when analyzed directly using titration, gave NaCl values of 3.8% and 6.5%, with a bias of 26.7% and 8.3%, respectively. In contrast, when the pH of the brines before analysis was corrected to 6.5, the titration results were 3.0% and 6.2%, with a bias of 0% and 3.0%, respectively (Table 1). The same results were recorded when the same brine samples were analyzed with a refractometer.

### 3.2. Long-Term Processing Tests Under spCO_2_ Conditions

The debittering tests under spCO_2_ conditions were studied using brines acidified with 0.5% (*w v*^−1^) citric acid and a NaCl concentration ranging from 0% to 6% (*w v*^−1^) NaCl. The traditional two-phase processing method (debittering and packaging) was applied by incubating the olives in brine in hermetically sealed demijohn bottles for 10 months, followed by packaging in glass jars and pasteurization. The single-phase processing method was instead applied to the same olives in brine, incubated in tightly closed glass jars for the same period of time without resorting to subsequent packaging but only to final pasteurization.

#### 3.2.1. Changes at the Onset of Incubation

**Figure 2:**
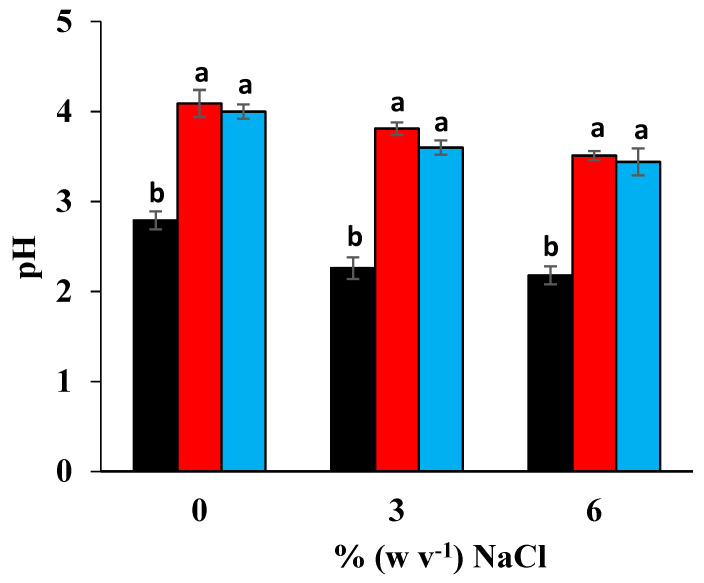
The pH of the brine used at the beginning of the trials (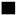) and after 15 days of fermentation of table olives with the two-phase (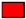) and single-phase (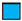) methods in the presence of spCO_2_. Histograms with different letters are significantly different at *p* ≤ 0.05.

The olive brine is initially contaminated by the microbiota adhering to the surface of the olives and by that present in the industrial environment where the resident yeast biota (e.g., in fermentation vessels) has significant contribution in fermentation [24]. Through the more or less stringent stress conditions created in the brine by the pH, salt, and spCO_2,_ it is possible within the first hours of incubation to control the microbiological risks and prevent the spoilage of the olives [25]. The chemical analyses of brine samples taken after 24 h and 15 days of incubation of the olives processed under spCO_2_ indicated a strongly acidic pH lower than 3 in the first hours of incubation, while in the following days, the buffering capacity of the raw product [26] stabilized the pH at around 4 (Figure 2).

**Figure 3:**
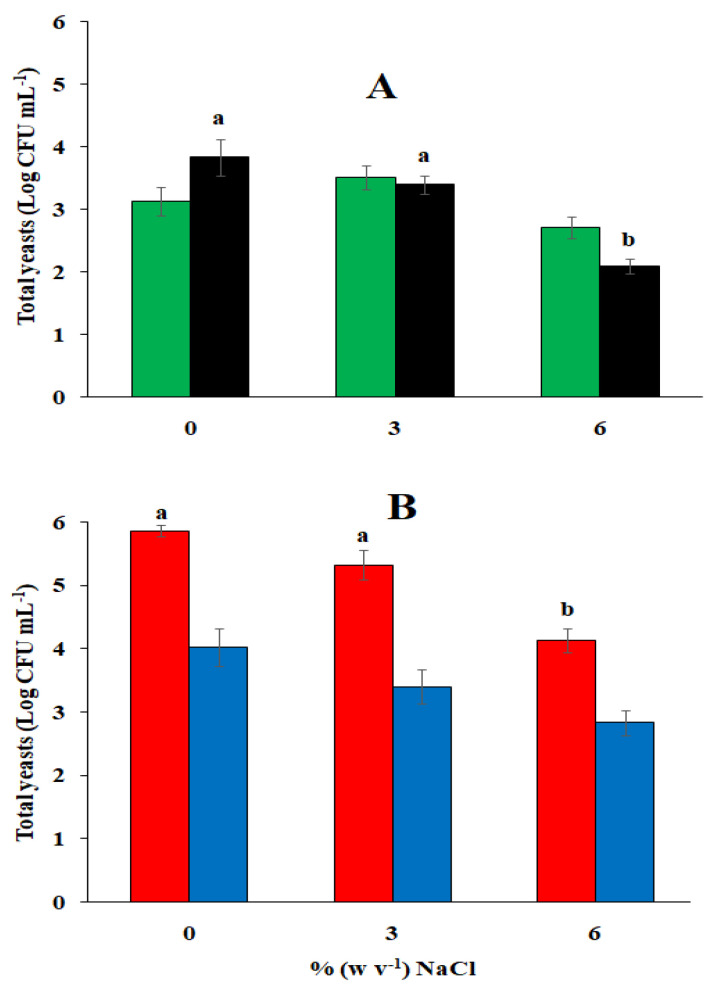
The microbiological analysis of brine after 24 h (**A**) and 15 days of incubation under spCO_2_ conditions (**B**). The mean number of yeasts detected in brine after 24 h of fermentation under spCO_2_ conditions with the two processing methods (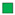). The mean number of molds detected after 24 h of fermentation with the two processing methods (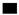). The total number of yeasts detected in the brine after 15 days of fermentation of table olives with the two-phase method (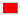) and the single-phase method (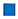). Histograms with different letters are significantly different at *p* ≤ 0.05.

**Figure 4:**
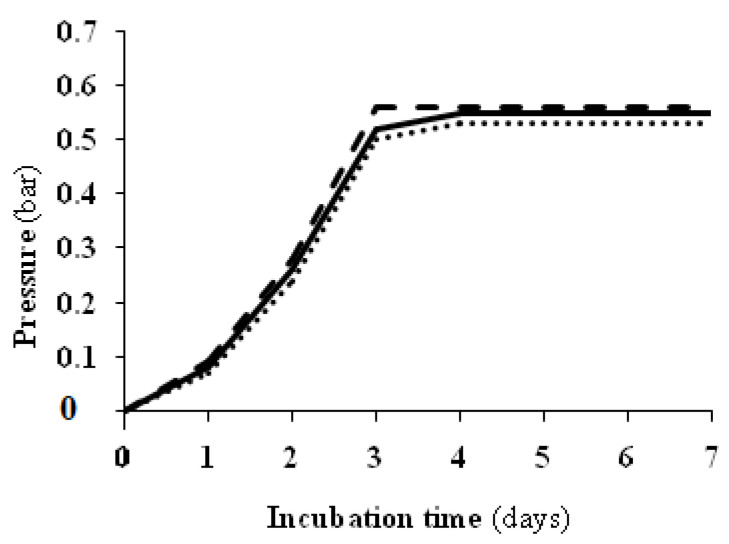
Increased pressure in demijohn bottles with table olives processed with the two-phase method in the first days of incubation. (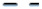), 0% NaCl; (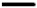), 3% (*w v*^−^^1^) NaCl; (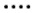), 6% (*w v*^−1^) NaCl.

Microbiological analyses performed on the same brine samples after 24 h of incubation in demijohn bottles (two-phase method) and glass jars (single-phase method) indicated the absence of all bacterial groups investigated with the selective media reported in the Section 3. The size of the yeast population ranged from 2.9 Log CFU per mL to 3.7 Log CFU per mL in the brine with varying NaCl contents. On the contrary, the mold, unlike the bacteria, better tolerated the acidic pH of the brine with 3% (*w v*^−1^) NaCl at the beginning of the incubation. Their number showed significant differences (*p* ≤ 0.05) and varied on average from a minimum of 2.10 Log CFU per mL of brine with 6% (*w v*^−1^) NaCl to a maximum of 3.9 Log CFU per mL in the brine with 0% (*w v*^−1^) NaCl (Figure 3A). The results shown in Figure 3A are in agreement with those reported in Figure 1, where TAB isolated from olives initially suffered a strong reduction in the acidified brine. After 15 days of incubation, microbiological analyses highlighted the disappearance of molds in all brines and the presence of yeasts alone, which dominated the entire fermentation process with a fair range of biodiversity. In our previous findings [13], it was demonstrated that spCO_2_ is very active in controlling bacteria in the brine. In this case, however, the initial bacterial reduction is mainly attributable to the acid pH since the spCO_2_, self-produced by the brine in the demijohn bottles, reached significant levels only after the third day of incubation (Figure 4). Microbiological analyses performed after 15 days of incubation under spCO_2_ conditions highlighted an increase in the amount of yeast only in the brines with reduced salt content incubated in the demijohn bottles in agreement with the two-phase processing method. However, even if it was generally observed across all the samples examined that the growth of yeasts, under spCO_2_ conditions, was reduced by the increase in the concentration of NaCl, significant differences (*p* ≤ 0.05) were recorded in the brines of the demijohns bottles provided with 0% and 3% (*w v*^−1^) NaCl (Figure 3B). These results are in agreement with our previous findings [16]. The lower yeast concentration recorded in the brines incubated in glass jars with the single-phase processing method, also considering the other data reported below, could be attributed to the different concentrations of free CO_2_ and the level of spCO_2_ initially reached in the two types of containers.

#### 3.2.2. Dynamics of Microbial Counts in Brine During Incubation

Evolution dynamics of the main microbial groups (bacteria, yeasts, and molds) studied in the first five months of incubation highlighted the absence of bacteria and molds in the brines of demijohn bottles and glass jars. The concentration of yeasts in the brines was influenced by the salt content to a greater extent in glass jars than in demijohn bottles. Table 2 reports the dynamics of the yeast content in the brines incubated with the two methods, in demijohn bottles and glass jars, recorded in the first five months of incubation. The brine from demijohn bottles of the two-phase method was richer in yeasts than that from glass jars of the single-phase method. As shown in Table 2, in the brine samples taken monthly from demijohn bottles, the number of yeasts ranged from a minimum of 4.76 Log CFU mL^−1^ to a maximum of 6.26 Log CFU mL^−1^ in the brine with 0% and 3% (*w v*^−1^) NaCl, while it was significantly reduced in the brine with 6% (*w v*^−1^) NaCl, ranging from a minimum of 2.01 Log CFU mL^−1^ to a maximum of 3.10 Log CFU mL^−1^. On the contrary, in the glass jars (single-phase processing method), the control of the number of yeasts was much more stringent even when the brine did not contain NaCl. In fact, the concentration of yeasts in the brine with 3% (*w v*^−1^) NaCl of the glass jars was significantly reduced compared to the control with 0% (*w v*^−1^) NaCl in the first five months of incubation, while in the samples with 6% (*w v*^−1^) NaCl, the recorded values were lower than 10 CFU mL^−1^ (Table 2). The microbiological analyses performed in the sixth month of incubation, in agreement with other studies, showed high similarity between brine and flesh microbiota in all samples examined [27]. However, the concentration of yeasts both in the olive flesh and in the brine was lower the higher the dose of NaCl used. More consistent results were obtained with the concentration of 6% (*w v*^−1^) NaCl, which, compared to the control without salt, reduced the number of yeasts by 55% and 100%, respectively, in the brine of black table olives processed with the two-phase method and in that of the same olives processed with the single-phase method (Table 3). Finally, the microbiological analysis performed at the end of the 10-month incubation period confirmed the greater presence of yeasts in the samples taken from the demijohn bottles used in the two-phase method compared to those from the glass jars of the single-phase method. However, unlike the previous analyses, a slight increase in the number of yeasts was recorded in both types of samples, which, however, was lower in the samples from the glass jars compared to those from the demijohn bottles (Table 3). The results reported in Table 2 and Table 3 confirm the synergistic antimicrobial activity, produced by the levels of spCO_2_ and salt content, against many microorganisms, including yeasts [13,15]. The greater presence of yeasts found in the brine samples from the demijohn bottles, compared to those from the glass jars, can likely be attributed to the different level of spCO_2_ reached in the two types of containers. In fact, in the demijohn bottles, where the pressure was regulated at 0.5 bar using special devices, the spCO_2_ reached a lower level than that recorded in the tightly closed glass jars. This hypothesis is supported by the higher concentration of free CO_2_ found in the brine of the glass jars used in the single-phase method reported in Table 4. The increase in the number of yeasts recorded in the last months of storage can be attributed to the increase in the external incubation temperature during the last four summer months.

#### 3.2.3. Yeast Biota

The yeast biota of table olives processed with the two-phase method in demijohn bottles and glass jars used in the single-phase processing method varied with the NaCl content, incubation period, and type of container. In the first five months of incubation, the following species were found: *Candida boidinii*, *Kloeckera apiculata*, *Nakazawaea molendinolei*, *Pichia manshurica*, *Saccharomyces cerevisiae* and *Zygotorulaspora mrakii*. In the following months of incubation, the disappearance of *K. apiculata* was noted. From Table 2, it is possible to note that in the first 5 months of fermentation in demijohn bottles with the two-phase method, the brine with 3% (*w v*^−1^) NaCl favored the growth of the species *C. boidinii* and *Z. mrakii* in a manner similar to the control without salt, although with a different prevalence ratio between them. In contrast, in the brine with 6% (*w v*^−1^) NaCl, except for the first month of incubation, only the presence of the yeast species *C. boidinii* was observed. Instead, in the brine of table olives processed with the single-phase method, supplied with 0% and 3% (*w v*^−1^) NaCl in addition to the yeast species *C. boidinii*, *K. apiculata*, *N. molendinolei*, and *Z. mrakii*, described above, the presence of the yeast species *S. cerevisiae* was observed, which predominated at the second and third month of incubation in the free-salt control and at the third and fourth month in the brine with 3% (*w v*^−1^) NaCl (Table 2). Microbiological analyses of the samples collected after 6 and 10 months of incubation revealed the same yeast species, both in the brine and in the flesh. In the samples taken from the table olives processed in demijohn bottles with the two-phase method, as previously noted, the presence of the species *C. boidinii* and *Z. mrakii* was ascertained both in the brine and in the olive flesh with 0% (*w v*^−1^) NaCl, to which the species *P. manshurica* and *N. molendinolei* were added to the samples with 3% (*w v*^−1^) NaCl. Finally, in the samples with 6% (*w v*^−1^) NaCl, the species *C. boidinii* was associated with the species *N. molendinolei* and others in different proportions. On the contrary, in the samples from glass jars, only the presence of the yeast *C. boidinii* was noted in the brine with 0% (*w v*^−1^) NaCl and that of *S. cerevisiae* in the samples with 3% and 6% (*w v*^−1^) NaCl (Table 3). From the results shown in Table 2 and Table 3, it is evident that the most prevalent species spread during incubation were *C. boidinii*, *Z. mrakii*, and *S. cerevisiae*. The role of the yeasts *C. boidinii* and *Z. mrakii* in the fermentation of table olives has been examined by some authors for their benefits as contributors to the flavor of the fermented product [28,29]. The yeast *C. boidinii* is widespread in the brines of different cultivars of table olives. It can grow at low temperatures and tolerates high pH and a salt content higher than 10% (*w v*^−1^) NaCl [30]. It is a yeast known for its positive effects on the aroma of olives due to the production of esters [27]. The species *Z. mrakii* is a slow-growing yeast that, unlike the previous one, develops after 3 days on the MYGP agar medium. It is widespread in the brines of different cultivars of olives, including Leccino, Taggiasca, and Bosana [13,15,31,32]. The species *S. cerevisiae* has been reported to improve the quality and safety of naturally fermented table olives. It is known for its antioxidant activity, useful for protecting the fruits from the oxidation of unsaturated fatty acids and from the formation of peroxide [33,34]. The results, in agreement with previous studies, confirm that the levels of spCO_2_ and salt concentration strongly control the growth of the yeast species present in the brine. The presence of *S. cerevisiae* in the brine rich in free CO_2_ from olives processed with the single-phase method are in agreement with previous studies, where *S. cerevisiae* showed greater spCO_2_ tolerance [13].

#### 3.2.4. Technological Traits

The physicochemical characteristics of Leccino olives processed under spCO_2_ conditions in the presence of different salt contents showed significant (*p* ≤ 0.05) variations at the sixth and tenth months of incubation according to the NaCl concentration. The results of the physicochemical characteristics recorded after six and ten months of incubation (Table 4 and Table 5) agree with the different microbial contents shown in Table 2 and Table 3. In the samples processed with the two processing methods, a clear difference was noted between the physicochemical characteristics of the brine with 6% (*w v*^−1^) NaCl and those of the others with 0% and 3% *(w v*^−1^) NaCl. In detail, the pH remained close to the hygienic safety threshold (pH < 4.3) in all the analyzed brines with 6% (*w v*^−1^) NaCl [3]. The titratable acidity did not show substantial differences even if the average of the concentrations recorded in all the brine samples collected from the glass jars was generally higher. The concentration of NaCl in the olive flesh was lower than that of the brine, thus demonstrating that after six months of incubation, the osmotic equilibrium between the brine and the olive flesh has not yet been fully reached. On the contrary, after ten months of incubation, the equilibrium between the NaCl concentration of the brine and that of the olive flesh improved, and the maximum difference between the two concentrations decreased from 23% to 6%. In fact, at the end of ten months of incubation, in the samples debittered with both processing methods, maximum concentrations ranging from 1.60% to 3.21% (*w w*^−1^) were recorded in the flesh of the olives fermented under spCO_2_ in the presence of 3% and 6% (*w v*^−1^) NaCl, respectively (Table 5). The density varied from 1.020 to 1.040 g cm^−3^ in the brines with 0%, 3%, and 6% (*w v*^−1^) NaCl, while the free CO_2_ concentration was significantly higher in the brine from table olives processed in glass jars with the single-phase method, especially when the NaCl concentration reached 6% *(w v*^−1^) (Table 4 and Table 5). This high free CO_2_ content presupposes that in the glass jars, the spCO_2_ level was higher than the 0.5 bar set by the valves in the demijohn bottles of the two-phase processing method. From the comparison of the free CO_2_ concentrations shown in Table 4 and Table 5 with the results of the microbiological analyses shown in Table 2 and Table 3, it is clear that this parameter, directly linked to spCO_2_, is able to modulate the survival and growth of microorganisms in the brine of naturally fermented black table olives. The bitterness index (K_225_) recorded after six months of incubation varied in a similar way to the concentration of total polar phenolic compounds present in the same olive flesh. However, at this stage of fermentation, the concentration of total polar phenolic compounds present in the olive flesh of all the samples analyzed was higher than that highlighted in the respective brines. These differences varied from a minimum of 12% to a maximum of 30% in the brines taken from the demijohn bottles of the two-phase processing method. Instead, in the samples processed using the single-phase processing method, which were also richer in phenolic compounds, the differences were attenuated and ranged from 2% to 14% (Table 4). The chemical analyses carried out after 10 months of incubation compared with those of the sixth month showed a strong reduction in the bitterness index, especially in the samples from the glass jars (single-phase method), which showed a maximum reduction of 35%, against the maximum value of 13% recorded in the olive flesh from the demijohn bottles used in the two-phase processing method. Additionally, the concentration of phenols present in the olive flesh, with the exception of one case, decreased in the last months of incubation. At the tenth month of incubation, compared to the previous analysis carried out at six months, a reduction in the phenolic content in the olive flesh was highlighted in all samples, varying from 5% to 25%. Instead, the concentration of total polar phenolic compounds present in the brines increased from 9% to 32% depending on the salt content and the origin of the samples. Finally, it should be emphasized that in the samples analyzed after ten months of incubation, contrary to the results recorded in the previous sampling of the sixth month, the concentration of the total phenols present in the olive flesh was lower than that of the brine and the reduction ranged from 0 to 36% (Table 5). The different contents of total polar phenols recorded at the end of the incubation of black table olives processed in demijohn bottles and in glass jars, as reported in Table 5, agree with the different contents of yeasts recorded in the same samples during fermentation (Table 2 and Table 3). In fact, the greater presence of yeasts and, therefore, their greater catabolic activity carried out in the brine with 0% (*w v*^−1^) NaCl are in agreeance with the reduced contents of phenolic compounds found in both the flesh and brine of the same samples (Table 5).

#### 3.2.5. Packaging and Pasteurization

**Figure 5:**
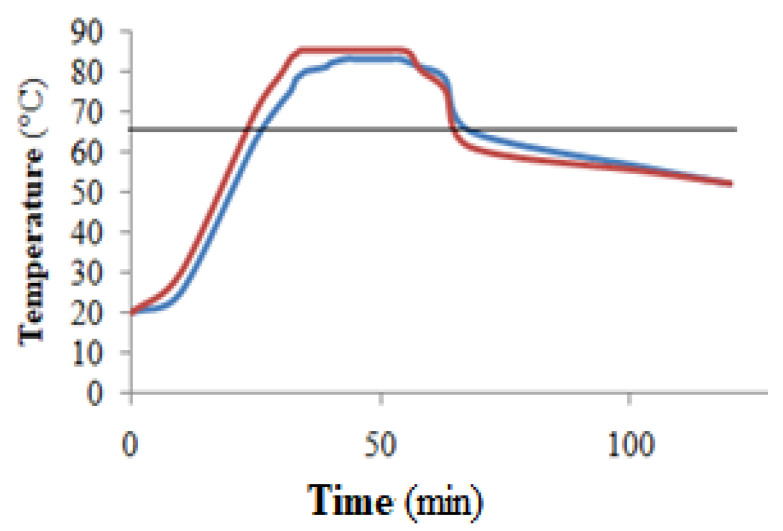
Pasteurization temperature dynamics. The mean (*n* = 3) temperature outside the glass jars (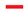); the mean (*n* = 3) temperature inside the glass jars (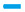).

Packaging corresponds to the second phase of the traditional two-phase natural-style black table olive processing method. The two-phase processing method is advantageous in that it allows for the processing of large masses of olives that, after debittering in barrels, can be stored for a long time, awaiting the second phase, in which they are packaged, pasteurized, and marketed. At the same time, however, it presents some problems related to high costs, the formation of undesirable microbial biofilm on the surface of the brines [16], pollution caused by the disposal of large masses of exhausted brines, and the reduction in biophenols in the olive flesh that migrate into the fresh brine used for packaging. On the contrary, the new single-phase processing method reported above, even if suitable for the processing of masses of olives significantly smaller than those processed with the two-phase processing method, could solve several of the problems outlined above. Before pasteurization, the brine of the packaged glass jars from the two-phase processing method showed a number of yeasts equal to 3.13 Log CFU mL^−1^ and the absence of other microorganisms; the microbial load in the glass jars used in the single-phase processing method was that reported in Table 3, relating to ten months of incubation. Subsequently, the glass jars with the olives from both the processing method were subjected to a single heat treatment of pasteurization at 85 °C for 20 min. Pasteurization is widely regarded as one of the most efficient methods for preserving food from pathogenic microorganisms, and a temperature of 85 °C allows this to be achieved with minimal negative effects on the quality of the table olives. This strategy is widely accepted as an internationally recognized standard for conforming to heat treatment processes for unprocessed products, as outlined by the European Parliament and the Council of the European Union regulation on food hygiene [21]. Monitoring the temperature inside the glass jars subjected to pasteurization indicated an average difference of 3 °C between the applied external temperature of 85 °C and that reached inside the glass jars. As reported in Figure 5, during the 20 min of heat treatment, the internal temperature of the glass jars did not exceed 82 °C. However, it should be noted that a temperature above 65 °C causes the aggregation of bacterial proteins, with the consequent death of the cells of many mesophilic microorganisms. The operating conditions used in the study allowed the samples to be exposed to a maximum temperature of 82 °C for 20 min and a temperature above 65 °C for 45 min (Figure 5). Microbiological analyses of pasteurized naturally fermented black table olives, in agreement with other studies [22], indicated the absence of microorganisms in all samples examined.

### 3.3. Sensory Quality

Sensory scores of olives during incubation showed substantial differences according to the salt concentration, processing method, and incubation period. As expected from the chemical and microbiological analysis results reported above, sensory analysis values obtained from olives processed in the presence of 0% (*w v*^−1^) NaCl were clearly negative and therefore lower than those of all other samples analyzed. Negative sensorial scores regarding defects such as odor, abnormal fermentation, and mustiness were highlighted starting from the sixth month of incubation both in the samples debittered with the two-phase method from demijohn bottles and in those from glass jars used in the single-phase processing method (Table 6). These results can be explained considering the higher catabolic activity carried out by the high number of yeasts present in the brine with 0% (*w v*^−1^) NaCl during incubation, as highlighted in Table 2 and Table 3. The absence of bitterness in the fruits (Table 6) and the lower concentration of phenols found during incubation in the samples with 0% (*w v*^−1^) NaCl (Table 4 and Table 5) support the hypothesis reported above. The dose of 6% (*w v*^−1^) NaCl recorded the best score for the overall quality attribute, especially when the single-phase processing method was applied. However, the use of 3% (*w v*^−1^) NaCl also showed acceptable results regarding the presence of defects, which, in the case of the two-phase processing method, disappeared with packaging and pasteurization due to the renewal of the brine. With regard to saltiness, in the flesh of olives processed with the two-phase method and debittered in brine with 3% and 6% (*w v*^−1^) NaCl, after 10 months of incubation, values equal to 1.60% and 3.11% (*w w*^−1^) of NaCl, respectively, were observed (Table 5). In the final product, 2 months after pasteurization, the NaCl level increased from 2.24% to 3.28% (*w w*^−1^). In the olive flesh processed using the single-phase method, the NaCl content remained unchanged at 1.60% and 3.21% (*w w*^−1^) NaCl, respectively, since fresh brine with 6% (*w v*^−1^) NaCl was not used (Table 5). The slight increase in salt from 1.60% to 2.24% (*w w*^−1^) NaCl was appreciated by the panelists (Table 6). Therefore, ultimately, from a sensorial point of view, the brine with 3% *(w v*^−1^) NaCl is equally interesting since it produced slightly lower results than the one with 6% (*w v*^−1^) NaCl, especially in the case of the single-phase processing method. Nonetheless, higher acidification is likely needed to keep the pH within the hygienic safety limits.

## 4. Conclusions

This work describes, for the first time, the evolution of microorganisms associated with naturally fermented cv. Leccino black table olives under spCO_2_ conditions with low salt content following the common traditional two-phase processing method and a new single-phase processing method. The present research, performed using a brine acidified with 0.5% (*w v*^−1^) citric acid and enriched with increasing doses of salt, indicated the disappearance of bacteria and molds during the first 15 days of fermentation of the olives in brine under spCO_2_ conditions. In the following months of fermentation, even in the absence of salt, only yeasts predominated, reaching the maximum concentration in brines with 0% (*w v*^−1^) NaCl. In brines with 3% and 6% (*w v*^−1^) NaCl, the number of yeasts was lower. This effect was more evident in the samples processed with the single-phase processing method, where the higher concentration of free CO_2_ present in the brine limited the number of yeasts below 10 CFU mL^−1^ in brine with 6% (*w v*^−1^) NaCl. The high presence of yeasts found in the brine with 0% (*w v*^−1^) NaCl compromised the physicochemical and sensorial characteristics of the final product, resulting in the appearance of some defects (Table 6). The final product concerning the olives processed with 3% (*w v*^−1^) NaCl using both methods of debittering was rated as having overall quality; however, the acidification of the brine was not sufficient to contain the pH values below the safety limit (pH < 4.3) during the entire fermentation process. The best debittering results were recorded with the olives debittered in the presence of 6% (*w v*^−1^) NaCl according to the single-phase processing method, where the higher concentration of free CO_2_ limited the number of yeasts. The new single-phase processing method tested in this study offers several advantages; however, despite the potential for developing new preparations, unlike the first method it does not allow for large masses of table olives to be processed.

## Figures and Tables

**Table 1:** Effects of brine acidification on NaCl values produced using AgNO_3_ titration.

Brine Sample	Unmodified Sample Analysis	Bias (%)	Neutralized Sample Analysis	Bias (%)
3% (*w v*^−1^) NaCl pH 6.5	3.0 ± 0.01 ^d^	0	-	-
3% (*w v*^−1^) NaCl acidified	3.8 ± 0.3 ^c^	26.7	3.0 ± 0.0	0
6% (*w v*^−1^) NaCl pH 6.5	6.1 ± 0.1 ^b^	1.67	-	-
6% (*w v*^−1^) NaCl acidified	6.5 ± 0.2 ^a^	8.3	6.2 ± 0.2	3.0

Mean ± standard deviation (*n*. repetitions = 3). Means in columns with different superscript letters are significantly different at *p* ≤ 0.05.

**Table 2:** Yeast growth during five months of incubation in the brines of black table olives processed with the two-phase and single-phase methods in the presence of 0%, 3%, and 6% *(w v*^−1^) NaCl and 0.5% (*w v*^−1^) citric acid under spCO_2_ conditions.

	Two-Phase Processing Method						Single-Phase Processing Method					
	0% NaCl		3% NaCl		6% NaCl		0% NaCl		3% NaCl		6% NaCl	
Month	Total Yeasts (Log CFU mL^−1^)	Preeminent Yeast Species (%)	Total Yeasts (Log CFU mL^−1^)	Preeminent Yeast Species (%)	Total Yeasts (Log CFU mL^−1^)	Preeminent Yeast Specie (%)	Total Yeasts (Log CFU mL^−1^)	Preeminent Yeast Species (%)	Total Yeasts (Log CFU mL^−1^)	Preeminent Yeast Species (%)	Total Yeasts (Log CFU mL^−1^)	Preeminent Yeast Species (%)
								*C.b.* (68)		Others (71)		
1	5.00 ± 0.10 ^a^	*C.b.* (72)	4.76 ± 0.86 ^a^	*C.b.* (60)	3.10 ± 0.97 ^b^	*P.m.* (50)	4.69 ± 0.50 ^a^	Others (30)	3.63 ± 0.81 ^b^	*Z.m.* (26)	0	n.d.
		*Z.m.* (28)		*Z.m.* (40)		*Z.m.* (50)		*Z.m.* (2)		*N.m.* (3)		
2	5.04 ± 0.92 ^a^	*C.b.* (62) *Z.m.* (38)	5.22 ± 0.48 ^a^	*Z.m.* (72) *C.b.* (28)	2.64 ± 0.25 ^b^	*C.b.* (100)	3.57 ± 0.70 ^ab^	*S.c.* (90) *C.b.* (10)	3.32 ± 0.54 ^b^	*K.a.* (52) *S.c.* (48)	1.31 ± 0.11 ^c^	n.d.
3	5.81 ± 0.03 ^a^	*Z.m.* (96) *C.b.* (4)	5.77 ± 0.04 ^a^	*Z.m.* (95) *C.b.* (5)	2.01 ± 0.09 ^c^	*C.b.* (100)	3.97 ± 0.49 ^b^	*S.c.* (70) *C.b.* (30)	2.50 ± 0.21 ^c^	*S.c.* (100)	0	n.d.
4	6.03 ± 0.21 ^a^	*Z.m.* (94) *C.b.* (6)	5.59 ± 0.15 ^a^	*Z.m.* (88) *C.b.* (12)	3.04 ± 0.10 ^b^	*C.b.* (100)	4.19 ± 0.23 ^ab^	*S.c.* (50) *C.b.* (50)	1.23 ± 0.09 ^c^	*S.c.* (100)	0	n.d.
5	6.26 ±	*Z.m.* (92)	5.40 ±	*Z.m.* (82)	3.08 ±	*C.b.* (100)	4.39 ±	*C.b.* (80)	0	n.d.	0	n.d.
	0.60 ^a^	C.b. (8)	0.28 ^a^	*C.b.* (18)	0.11 ^b^		0.15 ^ab^	*S.c.* (20)				

Below the detection limit of 10 CFU mL^−1^. n.d., not detected. C.b., Candida boidinii: K.a., Kloeckera apiculata; P.m., Pichia manshurica; Z.m., Zygotorulaspora mrakii; N.m., Nakazawaea molendinolei; S.c., Saccharomyces cerevisiae. Mean ± standard deviation (n. repetitions = 3). Values in lines with different letters are significantly different from each other at *p* ≤ 0.05.

**Table 3:** Yeast growth at the sixth and tenth months of incubation in the brines of black table olives processed with the two-phase and single-phase methods in the presence of 0%, 3%, and 6% (*w v*^−1^) NaCl and 0.5% (*w v*^−1^) citric acid under spCO_2_ conditions.

		Two-Phase Processing Method						Single-Phase Processing Method					
		0% NaCl		3% NaCl		6% NaCl		0% NaCl		3% NaCl		6% NaCl	
Months		Total Yeasts (Log CFU mL^−1^)	Preeminent Yeast Species (%)	Total Yeasts (Log CFU mL^−1^)	Preeminent Yeast Species (%)	Total yeasts (Log CFU mL^−1^)	Preeminent Yeast Species (%)	Total Yeasts (Log CFU mL^−1^)	Preeminent Yeast Species (%)	Total Yeasts (Log CFU mL^−1^)	Preeminent Yeast Species (%)	Total Yeasts (Log CFU mL^−1^)	Preeminent Yeast Species (%)
6	Brine	6.13 ± 0.38 ^a^	*Z.m.* (87) *C.b.* (13)	5.30 ± 0.41 ^a^	*Z.m.* (40) *P.m.* (20) *C.b.* (25) *N.m.* (15)	2.86 ± 0.11 ^b^	*C.b.* (50) *N.m.* (50)	4.41 ± 0.22 ^ab^	*C.b.* (100)	0	n.d.	0	n.d.
	Flesh	5.13 ± 0.48 ^a^	*Z.m.* (84) *C.b.* (14) *C.d.* (2)	4.42 ± 0.41 ^b^	*Z.m.* (64) *C.b.* (16) *P.m.* (12) *N.m* (8)	2.38 ± 0.05 ^c^	*C.b.* (50) *N.m.* (50)	3.70 ± 1.20 ^bc^	*C.b.* (100)	0	n.d.	0	n.d.
10	Brine	6.30 ± 0.05 ^a^	*C.b.* (100)	5.68 ± 0.14 ^a^	*C.b.* (98) *Z.m.* (2)	4.28 ± 0.07 ^b^	*C.b.* (98) Others (2)	4.10 ± 0.15 ^b^	*C.b.* (100)	3.68 ± 0.23 ^bc^	*S.c.*(100)	3.13 ± 0.20 ^c^	*S.c.*(100)
	Flesh	4.51 ± 0.31 ^a^	*C.b.* (100)	4.34 ± 0.33 ^ab^	*C.b.* (78) *N.m.* (20) *Z.m.* (2)	4.48 ± 0.54 ^ab^	*C.b.* (100)	5.24 ± 0.10 ^a^	*C.b.* (100)	3.67 ± 0.26 ^b^	*S.c.* (80) *C.b.* (20)	3.74 ± 0.17 ^b^	*S.c.* (100)

Below the detection limit of 10 CFU mL^−1^. n.d., not detected. Z.m., Zygotorulaspora mrakii; C.b., Candida boidinii: C.d., Candida diddensiae. P.m., Pichia manshurica; N.m., Nakazawaea molendinolei; S.c., Saccharomyces cerevisiae. Mean ± standard deviation (n. repetitions = 3). Means in lines with the different superscript letters are significantly different at *p* ≤ 0.05.

**Table 4:** Physicochemical characteristics of black table olives processed for six months with the two-phase and single-phase methods in the presence of 0%, 3%, and 6% (*w v*^−1^) NaCl and 0.5% (*w v*^−1^) citric acid under spCO_2_ conditions.

Parameters	Two-Phase Processing Method						Single-Phase Processing Method					
	0% NaCl		3% NaCl		6% NaCl		0% NaCl		3% NaCl		6% NaCl	
	Brine	Flesh	Brine	Flesh	Brine	Flesh	Brine	Flesh	Brine	Flesh	Brine	Flesh
pH	4.60 ± 0.57 ^a^	4.63 ± 0.10 ^a^	4.56 ± 0.01 ^a^	4.82 ± 0.25 ^a^	4.20 ± 0.49 ^b^	4.37 ± 0.40 ^b^	4.65 ± 0.01 ^a^	5.12 ± 0.40 ^a^	4.55 ± 0.04 ^a^	4.61 ± 0.30 ^a^	4.30 ± 0.04 ^b^	4.42 ± 0.30 ^b^
Titratable acidity (% citric acid)	0.46 ± 0.11	n.d.	0.50 ± 0.04	n.d.	0.52 ± 0.02	n.d.	0.69 ± 0.01	n.d.	0.69 ± 0.04	n.d.	0.68 ± 0.01	n.d.
NaCl (%, *w v*^−1^)	0	0	1.82 ± 0.08 ^b^	1.64 ± 0.33 ^b^	3.12 ± 0.35 ^a^	2.39 ± 0.47 ^ab^	0	0	1.65 ± 0.13 ^b^	1.58 ± 0.04 ^b^	3.45 ± 0.08 ^a^	2.99 ± 0.03 ^ab^
Density (g cm^−3^)	1.020 ^c^	n.d.	1.030 ^b^	n.d.	1.040 ^a^	n.d.	1.020 ^c^	n.d.	1.030 ^b^	n.d.	1.040 ^a^	n.d.
Free CO_2_ (g Kg^−1^)	1.318 ± 0.04 ^c^	n.d.	1.098 ± 0.06 ^d^	n.d.	1.199 ± 0.03 ^c^	n.d.	1.837 ± 0.09 ^b^	n.d.	1.940 ± 0.07 ^b^	n.d.	2.996 ± 0.09 ^a^	n.d.
Bitterness (K_225_)	n.d.	2.247 ± 0.485 ^b^	n.d.	3.256 ± 0.077 ^ab^	n.d.	3.785 ± 0.221 ^a^	n.d.	3.253 ± 0.141 ^ab^	n.d.	4.029 ± 0.088 ^a^	n.d.	4.392 ± 0.412 ^a^
Total polar phenols (mg CAE g^−1^)	1.70 ± 0.0 ^c^	1.92 ± 0.42 ^c^ (12%) ^1^	2.53 ± 0.04 ^b^	3.07 ± 0.05 ^ab^ (17%)	2.41 ± 0.03 ^b^	3.43 ± 0.07 ^a^ (30%)	2.50 ± 0.12 ^b^	2.66 ± 0.01 ^b^ (6%)	3.44 ± 0.35 ^a^	3.51 ± 0.16 ^a^ (2%)	3.25 ± 0.11 ^ab^	3.77 ± 0.40 ^a^ (14%)

n.d., not detected. CAE, caffeic acid equivalent. ^1^ Difference compared to the brines. Mean ± standard deviation (*n*. repetitions = 3). Values in lines with different letters are significantly different from each other at *p* ≤ 0.05.

**Table 5:** Physicochemical characteristics of black table olives processed for ten months with the two-phase and single-phase methods in the presence of 0%, 3%, and 6% (*w v*^−1^) NaCl and 0.5% (*w v*^−1^) citric acid under spCO_2_ conditions.

Parameters	Two-Phase Processing Method						Single-Phase Processing Method					
	0% NaCl		3% NaCl		6% NaCl		0% NaCl		3% NaCl		6% NaCl	
	Brine	Flesh	Brine	Flesh	Brine	Flesh	Brine	Flesh	Brine	Flesh	Brine	Flesh
pH	4.58 ± 0.04 ^a^	4.64 ± 0.09 ^a^	4.60 ± 0.01 ^a^	4.70 ± 0.11 ^a^	4.32 ± 0.02 ^b^	4.50 ± 0.01 ^ab^	4.86 ± 0.03 ^a^	5.10 ± 0.08 ^a^	4.55 ± 0.01 ^ab^	4.71 ± 0.11 ^a^	4.35 ± 0.02 ^b^	4.54 ± 0.12 ^ab^
Titratable acidity (% citric acid)	0.51 ± 0.01	n.d.	0.53 ± 0.02	n.d.	0.50 ± 0.01	n.d.	0.65 ± 0.03	n.d.	0.62 ± 0.02	n.d.	0.70 ± 0.01	n.d.
NaCl (%, *w v*^−1^)	0	0	1.70 ± 0.37 ^b^	1.60 ± 0.09 ^b^	3.00 ± 0.04 ^a^	3.11 ± 0.11 ^a^	0	0	1.50 ± 0.08 ^b^	1.60 ± 0.14 ^b^	3.32 ± 0.10 ^a^	3.21 ± 0.08 ^a^
Density (g cm^−3^)	1.020 ^c^	n.d.	1.030 ^b^	n.d.	1.040 ^a^	n.d.	1.020 ^c^	n.d.	1.030 ^b^	n.d.	1.040 ^a^	n.d.
Free CO_2_ (g Kg^−1^)	1.546 ± 0.02 ^c^	n.d.	1.297 ± 0.08 ^c^	n.d.	1.310 ± 0.07 ^c^	n.d.	1.890 ± 0.10 ^b^	n.d.	2.040 ± 0.09 ^b^	n.d.	2.706 ± 0.09 ^a^	n.d.
Bitterness (K_225_)	n.d.	1.989 ± 0.028 ^b^ [−11%] ^1^	n.d.	2.949 ± 0.006 ^a^ [−9.5%]	n.d.	3.288 ± 0.017 ^a^ [−13%]	n.d.	2.103 ± 0.017 ^b^ [−35%]	n.d.	2.854 ± 0.015 ^a^ [−29%]	n.d.	3.036 ± 0.005 ^a^ [−31%]
Total polar phenols (mg CAE g^−1^)	1.94 ± 0.0 ^b^ [14%]	1.94 ± 0.08 ^b^ (0%) ^2^ [1%]	3.15 ± 0.10 ^a^ [25%]	2.93 ± 0.28 ^ab^ (−7%) [−5%]	3.19 ± 0.07 ^a^ [32%]	3.18 ± 0.03 ^a^ (0.31%) [−7%]	3.01 ± 0.11 ^b^ [20%]	1.93 ± 0.01 ^b^ (−36%) [−27%]	3.85 ± 0.14 ^a^ [12%]	2.65 ± 0.11 ^ab^ (−31%) [−25%]	3.58 ± 0.12 ^a^ [9%]	2.74 ± 0.08 ^ab^ (−24%) [−27%]

n.d., not detected. ^1^ Difference compared to the sixth month of incubation. CAE, caffeic acid equivalent. ^2^ Difference compared to the brine. Mean ± standard deviation (*n*. repetitions = 3). Values in lines with different letters are significantly different from each other at *p* ≤ 0.05.

**Table 6:** Sensory score attributes of naturally processed Leccino table olives with 0%, 3%, and 6% (*w v*^−1^) NaCl and 0.5% (*w v*^−1^) citric acid after 6 and 10 months of incubation under spCO_2_.

Gustatory and Olfactory Attributes	Two-Phase Processing Method			Single-Phase Processing Method		
	0% NaCl 1 ^1^ 2 ^2^ 3 ^3^	3% NaCl 1 2 3	6% NaCl 1 2 3	0% NaCl 1 2 3	3% NaCl 1 2 3	6% NaCl 1 2 3
Odor	1 ^4^ 1 2	2 2 3	3 3 3	1 1 1	3 3 3	4 4 4
Saltiness	1 1 2	2 2 3	3 3 3	1 1 1	2 2 2	3 3 3
Bitterness	4 4 4	3 4 4	3 4 4	4 4 4	3 4 4	2 4 4
Abnormal fermentation	1 1 2	3 3 4	4 4 4	1 1 1	3 3 3	4 4 4
Mustiness	1 2 2	3 3 4	4 4 4	1 2 2	3 3 3	4 4 4
Overall quality	1 1 2	3 3 4	4 4 4	1 1 1	3 3 3	4 4 4

^1^ Median after six months incubation; ^2^ median after ten months incubation; ^3^ median of the final pasteurized product. ^4^ Unsatisfactory: 1 point; moderate: 2 points; good: 3 points; excellent: 4 points.

## Data Availability

The original contributions presented in the study are included in the article; further inquiries can be directed to the corresponding author.
